# Commentary: Chinese Herbal Medicine Usage Reduces Overall Mortality in HIV-Infected Patients With Osteoporosis or Fractures

**DOI:** 10.3389/fphar.2022.870855

**Published:** 2022-05-24

**Authors:** Chun Zhang, Hongyan Li, Qianqian Niu, Yulan Xie, Jianhua Hu, Cuie Liu, Xiaofei Shang, Xiuhui Li

**Affiliations:** Beijing Youan Hospital, Capital Medical University, Beijing, China

**Keywords:** HIV/AIDS, traditional medicines, diagnosis, treatment, TCM

HIV remains a public health threat related to human rights and social justice issues; 38 million people are living with HIV (WHO, 2021). There is a large gap between that number and the globally agreed goal of less than 200,000 HIV deaths by 2030, as well as the new 95%–95%–95% targets (provide 95% of all people at risk with access to HIV combination prevention options, ensure that 95% of people living with HIV know their status, and get 95% to be on HIV treatment) to be reached by 2025 set by the UN General Assembly fifth high-level meeting on HIV and AIDS, especially for middle- and low-income countries (The Lancet Public Health, 2021). Considering that over 80% of the global population depends on herbal medicine for basic healthcare (Vines, 2004), Ho et al. (2021) recently reported that Chinese herbal medicine could reduce overall mortality in HIV-infected patients with osteoporosis or fractures. The impact of traditional medicine (TM) on HIV/AIDS has been receiving much attention in HIV research, testing, and care, which was defined as health practices, approaches, knowledge, and beliefs incorporating plant, animal, and mineral-based medicines, spiritual therapies, manual techniques, and exercises, applied singularly or in combination to treat, diagnose, and prevent illnesses or maintain wellbeing (Fokunang et al., 2011).

Delay of HIV testing and diagnosis is common in Africa, where 62% of 517 newly diagnosed HIV patients had consulted a traditional healer prior to a health facility (Audet et al., 2014), and traditional healers (including herbalists, spiritualists, diviners, or any other practitioner trained or gifted in these forms of healing and recognized as such by the community) had a positive impact on HIV testing (UNAIDS, 2006; Sundararajan et al., 2021). Compared with a control group, traditional healers increased the likelihood of receiving an HIV test 4.4 times in sub-Saharan Africa, and clients newly diagnosed with HIV had higher rates of ART initiation (Sundararajan et al., 2021). Traditional healers may be an effective strategy to improve the HIV care cascade and decrease mortality in Senegal (Benzekri et al., 2019), and incorporating traditional healers into HIV care could improve HIV-related outcomes. The Chinese government has been establishing powerful TCM-based healthcare, which would help screen and manage many diseases, such as COVID-19, SARS, viral influenza, and infectious hepatitis and could provide more assistance for HIV/AIDS.

During the past decades, due to intolerable adverse effects, cross-resistance, personal prejudice or misconceptions, and poor compliance with ART, HIV/AIDS patients have been increasingly seeking help from TM. Although there are no high-quality, well-designed clinical trials to prove the safety and efficacy of TM for preventing HIV, approximately 17.6%–64% of individuals with HIV/AIDS in Africa are treated with TM, which remains part of the cultural framework for spiritual and psychosocial support (Boum et al., 2021). In the United States, approximately 35%–75% of individuals with HIV/AIDS use TM to treat HIV-related health concerns. The integration of TM and Western medicine in HIV has become a feasible idea and should be given more attention.

China has made tremendous progress in HIV-1 control with the scale-up of HIV testing and treatment (Wang et al., 2013). In 2004, the National Administration of Traditional Chinese Medicine (TCM) started a national TCM HIV treatment trial program (NTCMTP) that provided free TCM to patients living with HIV in 28 provinces. Long-term treatment with TCM enhanced their quality of life and reduced morbidity and mortality, with earlier TCM associated with a greater benefit, and integrating TCM and ART is recommended. During the asymptomatic stage, TCM physicians prescribe formulas to stimulate the defense mechanism to fend off illness, aiming to maintain and enhance immune function to delay disease progression. During the AIDS stage, TCM focuses on relieving symptoms and AIDS-related opportunistic infections. The number of HIV/AIDS patients treated with free TCM reached 60,000 in 2021.

Liu et al. (2019) reported that ART integrated with TCM raised or maintained CD4 counts regardless of the baseline level and lowered various AIDS-induced complications in 802 patients by 78% over 4 years. A study of the effect of TCM on overall mortality in HIV patients with osteoporosis or fractures in the Taiwan area showed that TCM users had much lower mortality (hazard ratio = 0.43, *p* < 0.005) and higher survival (*p* = 0.004, log-rank test) rates (Ho et al., 2021). In addition, *Tangcao* tablets and *Qiankunning* capsules significantly reduced the HIV RNA level of patients more than that in the placebo group from 6 months to 1 year in two double-blind RCTs, respectively (Shi and Peng, 2003; Zhao et al., 2006). Meanwhile, the 13 RCTs reported that TCM was effective for oral candidacies, peripheral neuropathy, skin rash, diarrhea, and other complications. For example, Shenling Baizhu San (参苓白术散) contributes to HIV-related diarrhea, and Banxia Xiexin Tang (半夏泻心汤) has a promising gastrointestinal benefit (Wang and Zou, 2010). In our clinical practice funded by NTCMTP, there have been no cases of morbidity or mortality among 150 patients who received ART and TCM therapies in the past 5 years. Two men with HIV/AIDS accompanied by syphilis that both strongly opposed ART due to personal prejudices were each administered a single TCM in 2012; both lived stably with relatively normal levels of CD4^+^ counts and lower viral loads, and no side effects or complications were found.

The world faces many challenges in the AIDS response. HIV testing services in clinical and community settings should be improved, and all populations with HIV should benefit from better person-centered delivery of healthcare (Piot et al., 2015). As Boum and colleagues (2021) commended, it is necessary to integrate TM as a catalyst for improving healthcare quality and avoid patients “eating” herbal medicine without any regulation. For HIV testing, traditional healers could help advance the newly diagnosed rates and advocate for HIV/AIDS patients receiving ART. For most patients living with HIV, TM could be used as an adjuvant or alternative therapy to enhance the quality of life and reduce side effects, complications, and cross-resistance of ART. Considering that the global ART coverage of people living with HIV had only reached 68.97% in 2020 (WHO, 2021), TM application in the clinic provides the availability and accessibility of basic therapy for HIV/AIDS to decrease mortality, particularly in vast rural and extreme poverty areas.
